# Harnessing heterosis and male sterility in soybean [*Glycine max* (L.) Merrill]: A critical revisit

**DOI:** 10.3389/fpls.2022.981768

**Published:** 2022-10-10

**Authors:** Ayyagari Ramlal, Aparna Nautiyal, Pooja Baweja, Rohit Kumar Mahto, Sahil Mehta, Bingi Pujari Mallikarunja, Roshni Vijayan, Shukla Saluja, Vijay Kumar, Sunil Kumar Dhiman, S. K. Lal, Dhandapani Raju, Ambika Rajendran

**Affiliations:** ^1^Division of Genetics, ICAR-Indian Agricultural Research Institute (IARI), Pusa Campus, New Delhi, India; ^2^Department of Botany, Institute of Science, Banaras Hindu University (BHU), Varanasi, Uttar Pradesh, India; ^3^Department of Botany, Deshbandhu College, University of Delhi, New Delhi, India; ^4^DBC i4 Center, Deshbandhu College, New Delhi, India; ^5^Department of Botany, Maitreyi College, University of Delhi, New Delhi, India; ^6^School of Biotechnology, Institute of Science, Banaras Hindu University (BHU), Varanasi, Uttar Pradesh, India; ^7^School of Agricultural Sciences, K. R. Mangalam University, Gurugram, Haryana, India; ^8^Division of Genetics, Regional Research Centre, ICAR-Indian Agricultural Research Institute (IARI), Dharwad, Karnataka, India; ^9^Regional Agricultural Research Station, Kerala Agricultural University, Pattambi, Kerala, India; ^10^Department of Botany, Sri Venkateswara College, University of Delhi, New Delhi, India; ^11^Department of Botany, Shivaji College, University of Delhi, New Delhi, India; ^12^Department of Botany, Kirori Mal College, University of Delhi, New Delhi, India; ^13^Division of Plant Physiology, ICAR-Indian Agricultural Research Institute (IARI), Pusa Campus, New Delhi, India

**Keywords:** heterosis, soybean, male sterility, genetic diversity, self-pollination, seed yield

## Abstract

Soybean is a predominantly self-pollinated crop. It is also one of the important oilseed legumes. Soybean is an excellent crop having industrial, traditional, culinary, feeding, and cultural roles. Genetic diversity in breeding programs is of prime importance as it ensures the success of any breeding by enhancing the outcomes and results of the plants. The phenomenon wherein the progeny exhibits greater biomass (yield) and a faster rate of development and fertility than its parents is referred to as heterosis. As of now, heterosis is mainly limited to the trait of seed yield and is considered the basis for the development of better (superior) varieties. Male sterility (MS) is extensively used for the production of seeds and the improvement of crops coupled with the traditional breeding programs and molecular technology. Therefore, deployment of MS and heterosis in breeding soybean could yield better outcomes. This review aims to focus on two aspects, namely, MS and heterosis in soybean with its scope for crop improvement.

## Introduction

Soybean [*Glycine max* (L.) Merrill, Fabaceae; 2n = 4x = 40] is a self-pollinated crop. It is a multifaced nutritional food crop with high amounts of proteins (40%), fats (20%), oil contents and as a medicinal crop (Hymowitz, 1970; Singh et al., 2001; Rodrigues et al., 2006; Medic et al., 2014; Rajendran and Lal, 2020; Rajendran et al., 2022; Ramlal et al., 2022). Soybean is a paleopolyploid which has resulted in the presence of more than one copy of a gene in about 75% of its genes and shows differential expression (Friedrichs et al., 2016). The occurrence of domestication of soybean from its wild relative *Glycine soja* Sieb. and Zucc. dated back to 3,000–5,000 years in China and the landraces are spread throughout the globe (Carter et al., 2004; Tavaud-Pirra et al., 2009). The duplicated genes in homologous regions might interact similarly with the heterozygous alleles at the single locus. In contrast, the unequal allelic expression has been observed in hybrids due to differences in gene regulation (Burton, 2011). The discovery of female fertile mutants and male sterility (MS) in soybean by Brim and Young (1971) led to the path for heterosis in soybean (Gadag and Upadhyaya, 1995). The concept of heterosis is of prime importance in agriculture which in turn is essential for the production of seeds (yield) (Pandini et al., 2002). Heterosis, or sometimes it is referred to synonymously as “hybrid vigor,” is defined as the superiority of F_1_ seeds in terms of developmental rate, viability, resistance to diseases, and yield over their parents (two or more) having different genetic constitutions (Fehr, 1987; Pandini et al., 2002; Wu et al., 2021) that is widely used in various agriculturally important crops like rice and maize, vegetables, and perennials (Vaillancourt et al., 1995; Arcade et al., 1996; Kopp et al., 2002; Marcelo et al., 2007; Chen et al., 2020; Wang et al., 2021; Yu et al., 2021; Zhang et al., 2021). The success of the development of hybrids in soybean largely depends on the magnitude and direction with which it is being produced. Breeding and varietal development explicitly depend on the extent of heterosis (Gadag and Upadhyaya, 1995). “Soybeans show little heterosis, when they are crossed. Similarly, there is little inbreeding depression soybean self-pollinate” (Pioneer Hi-Bred International, Inc.). The main criteria for the production of heterosis in soybean include seed yield and the ability to produce hybrid seeds on a large scale. The extent of success in any breeding program depends on the availability of genetic diversity of the crops (Manjarrez-Sandoval et al., 1997). A total of 156,849 germplasm collections are available in soybean (Carter et al., 2004). The genetic diversity is increased by the availability of germplasm accessions, which thereby enhances soybean breeding programs simultaneously preserving the rare alleles that contribute to unique collections of germplasm. Understanding the germplasm and genetic diversity in crops is important for development and is, therefore, important in determining effective strategies that can improve agronomic traits (Jo et al., 2021).

The genetic distance in crops can be assessed by two parameters, namely, restriction fragment length polymorphism-based genetic similarity (RFLP-GS) and coefficient of parentage (CP). RFLP is defined as the bands common among the two genotypes, while the CP (also referred to as co-ancestry) is the probability of a random allele of one genotype is similar to that of another one at the same locus (Manjarrez-Sandoval et al., 1997). The methods that can evaluate genetic similarity include the estimation of genetic variance and heterosis. Similarly, molecular markers like random amplified polymorphic DNA (RAPD) and simple sequence repeats (SSRs) along with morphological (phenotypic traits) and biochemical (isozymes) markers can also be used to evaluate the genetic distance (Corrêa et al., 1999; Brown-Guedira et al., 2000; Kachare et al., 2020). The genetic diversity in the Chinese cultivated soybean was studied using the CP by Cui et al. (2000), using morphological traits by Dong et al. (2004), and using SSR markers by Wang et al. (2006). Similarly, using molecular markers like RAPD, various groups have studied the genetic diversity in soybean; Doldi et al. (1997) analyzed RAPD, Brown-Guedira et al. (2000) used SSRs and RAPD for American accessions, Li and Nelson (2001) used RAPDs for evaluating diversity in accessions of South Korea, Japan, and China, and Mulato et al. (2010) utilized SSR and EST-SSR along with other reports. Interspecific and intervarietal hybridization in soybean has been carried out for a long back for the improvement and to introduce variations of desirable genes from the wild-type species of soybean. Agronomic traits such as characters like 100-seed weight, seeds per plant, days to maturity, pods bearing nodes per plant, protein and oil contents, and plant height have been found to be associated with heterosis as was better in the hybrids than the mid and better parents (Khan and Dar, 2009).

Whenever the male reproductive parts fail to undergo normal developmental processes and produce organs that are nonfunctional, aborted, or absent and fail to participate in sexual reproduction, that phenomenon is referred to as MS. The primary types of MS include genetic MS (GMS), cytoplasmic MS (CMS), and cytoplasmic nuclear (Saxena and Hingane, 2015). So far, in soybean, the following MS systems have been developed *via* spontaneous process [GMS and environment-sensitive MS (ESMS)] and mutagens (GMS) (Saxena and Hingane, 2015). There are various applications of MS in crop improvement and breeding programs, namely, population and hybrid breeding along with utilization in heterosis (Saxena and Hingane, 2015; Li et al., 2019). With the involvement and implementation of modern techniques that include DNA methylation, transcriptional regulation, and histone modification (epigenetics), the scope of heterosis is widened, thereby assisting in elucidating the molecular and genetic basis of heterosis (Song et al., 2020). Similarly, MS can be induced through molecular cloning, recombinant methods, and plant transformation (Li et al., 2019), even though the main problem in the breeding programs is the identification and selection of parents producing progeny having superior characters. Moreover, despite tremendous efforts, the extent of hybridity is still restricted to the F_1_ generation only and the explanation for this cause is still least understood. Therefore, this review aims to provide an overview of the heterosis and MS in soybean and their applications and potential scope in developing novel progenies having better qualities than either of the parents.

## Heterosis in soybean

Heterosis is a complex phenomenon that involves many quantitative genes from vegetative growth-related genes to flowering and biomass to genes that provide resistance and tolerance against different stresses (Baranwal et al., 2012; Ryder et al., 2019) and proved to be a beneficial approach for improving economically important traits in crops. Although the field of heterosis in soybean is not new, it still significantly lacks updated information on the mechanisms of epigenetics in soybean.

### History of the development of heterosis in soybean: Field to lab

Many studies carried out by Veatch (1930), Woodworth (1933), Weiss et al. (1947), Kalton (1948), Leffel and Weiss (1958), Caviness and Vathana (1968), and Weber et al. (1970) reported the occurrence of heterosis in all the quantitative characters of soybean (Chaudhary and Singh, 1974). Brim and Cockerham (1961) generated F_1_ seeds through hand pollination. In contrast, some groups like Campos (1979) reported positive seed yield and developed an association with its components, while other groups reported negative heterosis between the seed weight and seeds per pod (reviewed by Gadag and Upadhyaya, 1995). Notably, Veatch (1930) reported 35.5% average mid-parent heterosis for yield and Paschal and Wilcox (1975) reported 8% average high-parent heterosis (reviewed by Cerna et al., 1997). Kiang and Gorman (1983) showed that high-yielding lines that were identified from the populations were developed by crossing genotypes with significantly greater genetic distances than any two others drawn at random. The genetic distances were estimated for 12 polymorphic isozyme systems (markers) on 100 soybean cultivars of the USA. The correlation between isozyme genotypes and quantitative traits has also been determined in two interspecific crosses (Kiang and Gorman, 1983; Cerna et al., 1997). Soybean shows the presence of heterosis. Out of eight crosses, five crosses showed and yielded positive transgressive parents with average heterosis of 32.7% than the mid-parent values (Gai et al., 1984). Nelson and Bernard (1984) showed around 19% heterosis for the trait yield and predicted that parental characteristics and pedigree of parents are sufficient to comment on the heterosis (Nelson and Bernard, 1984). Gadag and Upadhyaya (1995) selected seven lines of soybean, namely, Monetta, Bragg, Hardee, KHSb-2, Local Black Soybean (LBS), DS-74-62, and SL-96, and were crossed in all the combinations possible. The heterosis percentage of the better parent was obtained from −19.3 to 31.2 for days to flowering, −5.5 to 8.0 for days to maturity, plant height from −57.5 to 40.2, −39.8 to 69.1 for primary branches, −43.7 to 110.1 for the number of pods, −14.8 to 16.7 for seeds per pod, −45.5 to 21.7 for 100-seed weight, −43.6 to 121.9 for grain yield, −10.8 to 12.2 for protein content, and −14.2 to 11.3 for oil content. A total of nine hybrids showed yield superiority over better parents and three of them were significantly high yielding than the best parent (Gadag and Upadhyaya, 1995). They also reported that grain yield was higher in the hybrid of Bragg × Monetta and resulted in 170.9% more than the mid-parent and 121.9% more than the better parent. They further concluded that the hybrid obtained from crossing KHSb-2 × DS-74-62 was found to be the most promising with the better parent for the traits like plant height, days for flowering, pods per plant, 15.4% more yield, and 2.9% more oil than the best parent (Gadag and Upadhyaya, 1995). According to Manjarrez-Sandoval et al. (1997), the CP of parents predicted genetic variance accurately in the five inbred populations of soybean (Manjarrez-Sandoval et al., 1997). Pandini et al. (2002) reported that the genotype MTBR-95-123800 has its own potential use per cross for high yield. Shuming et al. (2002) attempted 1,326 crosses and observed average heterosis of 6.8% for each trait over high parental values. Burton and Brownie (2006) reported that the occurrence of yield heterosis in F_1_ and F_2_ generations of the progeny obtained by crossing two inbred lines of soybean was due to either more number of genes (duplications) favor heterosis or it is a result of gene complementation. They also compared the results of the studies of Brim and Cockerham (1961) (Gadag and Upadhyaya, 1995; Burton and Brownie, 2006). Hybrids, namely, DSb 1 × MACS 201, DSb 1 × PK 472, LSb 3 × PK 472, and DSb 1 × PK 1029 showed positive heterosis for yield per plant over mid-parent and better parent, whereas hybrids obtained from LSb 3 × MACS201 for oil content and JS 90-41 × PK472 for protein content showed positive heterosis (Ramana and Satyanarayana, 2006). According to Khan and Dar (2009), for the two traits, namely, pod length and seeds per pod, none of the 11 hybrids showed positive heterosis. Hybrids such as Bragg × JS 335, Bragg × DS 2106, and RSC-4 × DS 2106 showed heterosis for the pods per plant. Therefore, it can be concluded that seeds per pod, pod length, and the number of pods per plant manifest heterosis in soybean (Khan and Dar, 2009). The other events are given in Table 1.

**TABLE 1:** Development of heterosis in soybean.

	Traits	Population	Remarks	References
	Yield, maturity date and lodging resistance	Tested F_1_; tested unselected F_2_, F_3_, F_4_, F_5_ bulks together with parents	The correlation between F_2_ bulk and F_5_ selected lines for 17 crosses was 0.209.	Weiss et al., 1947
	Qualitative traits	Tested F_1_ and bulks of F_2_, F_3_, F_4_, F_5_	The results indicated additivity is the principal factor among the crosses	Brim and Cockerham, 1961
	Protein and oil content	F_1_	No significant heterosis in mid-parents for protein or oil while 0.9–1.5% to oil for heterosis in the high parent.	Weber et al., 1970
	–	_–_	Positive values—26.1% for a group of hybrids	Chaudhary and Singh, 1974
	Seed yield	F_1_	Five out of 27 hybrids range between 13 and 19% from the superior parent	Nelson and Bernard, 1984
	Seed yield	F_1_	85% mid-parent heterosis in F_1_ progeny and 62% high parent heterosis	Burton, 1987
	–	F2	The heterosis increased as the differences in phenotypes of parents increased	Gizlice et al., 1993
				Weber et al., 1970
	Yield and agronomic traits	BC_1_F_1_	Tested using single (−59% to + 37%—mid parent and −66% to + 17%—high parent), three-way (−14% to +16%—mid and −25% to −5%—high parent) and backcrosses (−7% to +42% —mid parent and −16% to + 42%—high parent)	Ortiz-Perez et al., 2007
	Seed yield and number of pods per plant	–	Bragg × IC-118013 and JS-80-21 × IC-118319	Shanti et al., 2008
	Isoflavone	F_1_	The isoflavone content was additive in nature and can be affected by the wild types for breeding purposes	Bi et al., 2015
	Cytosine methylation and 12 traits	–	Jilin 47 (no. 19), EXP (no. 12), Jilin 38 (no. 3) and Yi 3 (no. 6)—parental lines; Jilin 38 × Yi 3 (3 × 6), Jilin 38 × EXP (3 × 12), Jilin 38 × Jilin 47 (3 × 19), Yi 3 × Jilin 38 (6 × 3), Yi 3 × EXP (6 × 12), Yi 3 × Jilin 47 (6 × 19), EXP × Jilin 38 (12 × 3), EXP × Yi 3 (12 × 6), EXP × Jilin 47 (12 × 19), Jilin 47 × Jilin 38 (19 × 3), Jilin 47 × Yi 3 (19 × 6), Jilin 47 × EXP (19 × 12)—hybrid lines. The met levels were lower in the middle parent than in the hybrids	Wang et al., 2018

## Generation of heterosis and male sterility in soybean

### Epigenetics

Recently, epigenetics, the science of expression changes through DNA methylation, utilization of non-coding RNA (ncRNA), and histone modifications, is used in plant breeding as well as in determining the performance of hybrids. Here, phenotypic variation is observed as a result of changes in nucleotide sequences but not due to gene functions. At present, this area is utilized in crop improvement and plant breeding sciences (Tsaftaris et al., 2008; Gallusci et al., 2017; Rajnović et al., 2020). Nakamura and Hosaka (2010) observed that the levels of methylation were significantly different in the parents and hybrid potatoes and also stated that homozygosity and heterozygosity of the methylated DNA are known to regulate heterosis and inbreeding depression, respectively. Wang et al. (2018) studied that the methylation-sensitive amplification polymorphism (MSAP) method using the capillary electrophoresis was used for the estimation of cytosine methylation in the 15-day post-emergence leaves of parental and hybrid lines and reported that hypermethylation can be used for increasing the stem diameter. In contrast, Sun et al. (2015) and Kawanabe et al. (2016) showed that there is a relationship between heterosis and methylation of DNA. Chen et al. (2022) showed that there is a significant difference in the differentially methylated sites in the reciprocal hybrid of soybeans in different combinations (Chen et al., 2022). Hence, other plausible alternatives can be explored.

### Clustered regularly interspaced short palindromic repeats-Cas

Some of the most popular genome-editing tools developed and used so far include the recently proposed method that uses the RNA, a guided approach for editing the target genome referred to as clustered regularly interspaced short palindromic repeats (CRISPR)-associated protein 9 (CRISPR-Cas9) nuclease system (Zhu et al., 2020). This technique is being utilized in soybean as well along with other agriculturally important crops like rice, wheat, and tomato in crop improvement programs (Chen et al., 2021). Recently, stable MS lines were produced using this method in soybean using the ABORTED MICROSPORES (*AMS*) homologs that aid in the production of pollen wall and help in the degradation of the tapetum. The *GmAMS1* produces MS lines, while *GmAMS2* fails to produce (Chen et al., 2021). This area can be explored further for the development of such MS lines in soybean by taking the cues from similar studies carried out using carbon starved anther (*CSA*) for the generation of photosensitive male-sterile lines in rice (Li J. et al., 2016), temperature-sensitive sterile lines in rice using the TMS5 gene by Zhou et al. (2016), *Ms1* knock-out in wheat, and the stamen-specific gene (*SlSTR1*) for tomato by Okada et al. (2019) and Du et al. (2020), respectively.

### Omics

The omics approaches include transcriptomics at the gene and transcriptional level, and proteomics at both the protein and translational levels along with high-throughput sequencing methods are emerging methodologies that are being used for the generation of MS and induction of heterosis in plants (Li et al., 2019; Li et al., 2022; Liu et al., 2022). The reports using the omics technology for harnessing heterosis and developing MS lines in soybean are scant. However, Zhang et al. (2017) observed that 681 and 899 genes were identified that are differentially expressed between the flowers of two hybrid soybeans, namely, HYBSOY-1 and HYBSOY-5, and their parents, respectively.

## Male sterility and its mechanism in soybean

### Male sterility in soybean

Male sterility in plants refers to a phenomenon where the reproductive structures either fail to develop or show reduced growth. These can be either of nuclear origin (nuclear/genetic; NMS) or mitochondrial genes (cytoplasmic; CMS) along with another MS system known as photoperiod/temperature-sensitive genic MS (PTGMS) which is also referred to as environment-sensitive genic MS (EGMS) (El-Namaky and van Oort, 2017; Li et al., 2019; Nadeem et al., 2021). There are various demerits associated with these systems of sterility. For instance, due to excessive inbreeding, the CMS system possesses low genetic variability and the ability of restoration of CMS is also less but can be used to induce disease traits. In contrast, the case of photoperiod/temperature-sensitive genic MS requires specific environmental conditions to induce either male or female sterility. Thus, it cannot be applicable to all places. Hence, NMS proves to be as compared to other systems of sterility and could be utilized during hybrid breeding programs (Li et al., 2019). Li et al. (2019) described the main NMS and CMS that have been developed in soybean from 1928 to 2019 and 1985 to 2016, respectively.

The first nuclear MS line was produced by Owen (1928), while the first cytoplasmic male sterile line was developed by Davis (1985). As of now, over 50 CMS and 30 NMS mutants have been identified in soybean (Li et al., 2019). Mutation-induced sterility in soybean is primarily of two types, namely, male sterile, female sterile (MS-FS) and male sterile, female fertile (MS-FF), wherein in the former one, both male and female reproductive systems get affected, while in the latter one, only the male reproductive system lost its function, while the less or a reduced effect is seen in the female. The MS-FF lines are used more commonly in research owing to their ability to set seeds. The sterile lines are greatly used in the breeding programs (to develop novel varieties through recombining genotypes) and to understand the genetics of microgametogenesis and microsporogenesis. There are many criteria for the development of hybrids in soybean such as (1) yield increases must be realized in the Fl generation, (2) regular supply of female parents for crossing must be there for the male-sterile soybean plants, and (3) the transfer of pollens occurs between male-sterile and male-fertile plants. Nelson and Bernard (1984) used the lines of MS-FF for the production of F_1_ for the assessment of heterosis (Graybosch and Palmer, 1988). There is no economical way for the production of F_1_ hybrid seeds due to the unavailability of a good CMS/NRS and adequate numbers of pollen vectors (Burton, 2011). The composition of nuclear and cytoplasmic genes confers the MS in soybean. In some cases, it is monogenic in nature while becoming polygenic when the same genotype of a nucleus interacts with different cytoplasmic genes. It has been observed that inter- and intra-allelic complementation also affects fertility restoration in soybean. In the case of soybean, both gametophytically and sporophytically controlled restoration genes were reported (Li et al., 2019).

### Molecular basis of male sterility

There are recent pieces of evidence of unraveling the genetic mechanisms of MS in soybean but still many of the questions have remained unanswered. The crucial developmental stages include stamen primordial formation to maturation into pollens and thus occurrence of pollination, wherein any InDel mutations will result in the failure of normal functioning and cause sterility in males. The abnormality in the development of male flower arises from the mutations in the mitochondrial genome that resulted in the cytoplasmic MS suppressing the restorer of fertility (rf) genes. *GmMADS28*, a flower-enriched region of the AGL9/SEP subfamily, is associated with the filament length, the number of floral organs, the release of pollens, and sterility in soybean. In contrast, other reports showed that CMS also arises from the *atp* gene that forms ATPase. Later on, it was observed that CMS genes contain some parts of ATPase genes that in turn disrupt ATP production and cause MS. Furthermore, *GmMF1* is also associated with causing MS. In contrast, there are two pairs of restorers of fertility, including *NJCMS1A* is linked with the linkage groups (LGs), namely, “M” (loci: Satt626) and “A1” (loci: Satt300) and *NJCMS2A* is linked with the LG, namely, “D2” (loci: Satt135) (Li et al., 2019). Due to the lack of availability of fine mapping of the loci involved in MS, the molecular mechanisms remain obscured (Zhao et al., 2019). A total of 94 sterile mutants were identified and three-line system-based CMS was also developed (Ray et al., 2003; Palmer et al., 2012; Zhao et al., 2019; Nadeem et al., 2021). Along with these, three CMS restorer loci and two independent locus Rf-m were fine-mapped (Wang et al., 2016; Zhao et al., 2019). Similarly, genes related to NMS have also been identified in soybean. The LGs containing *ms3* and *ms4* on LG-D1b and *ms1* and *ms6* on LG-F are found to be involved in nuclear-based MS in soybean (Nadeem et al., 2021) [refer to Nadeem et al. (2021) for more information], although the molecular and genetic mechanisms are yet to be elucidated in soybean (Li J. et al., 2016; Nadeem et al., 2021).

## Applications of heterosis and male sterility in soybean breeding

### Scope of heterosis in crop improvement

There have been many incidences of technological advances in the field of agriculture to meet the growing demands for more food. However, we are facing many issues in managing the needs of the growing population. With the advent of technological advancement to meet the increasing demands, the utilization of heterosis in agriculture has strengthened the backbone in the production of hybrids in the cross as well as self-pollinated crops (Li et al., 2019). With the over-application of conventional breeding methods, the non-fixable variations remain unutilized in crops like soybean which are self-pollinated (Sharma and Maloo, 2017). With the identification of intercrossing and MS lines in soybean, the efficiency of hybrids can be increased (Sharma and Maloo, 2017). Mutagenesis is yet another powerful tool and technique through which new varieties can be developed (reviewed by Holme et al., 2019; Viana et al., 2019; Ayyagari and Rajendran, 2021). The primary goal in the breeding programs is the selection of parents which can result in producing individuals with high genetic variability for a particular trait (Friedrichs et al., 2016). There are many drawbacks that are associated with traditional breeding, including manual cross-pollination being cumbersome, non-economical, and time-consuming method, whereas MS, pollination through agents (entomophily), and natural means are considered better in producing a large amount of yield (Perez et al., 2009).

### Utilization of male sterility

Soybean is a majorly self-pollinated crop (pseudocleistogamous) with a rare occurrence of cross-pollination (Lord, 1981; Fujita et al., 1997; Takahashi et al., 2001) which thereby makes less difficult for the chances of the occurrence of heterosis (Burton and Brownie, 2006). The aim of a breeder is to select the superior genotypes having the better qualities/traits. Sometimes, this is often difficult to obtain due to inbreeding depression which in turn causes a population bottleneck. The potential of MS lines can be explored in soybean. For instance, NMS lines *ms1*, *ms3*, *ms4*, and *ms6* including others have been utilized in improving agronomic traits like protein and oil content and yield, while some have developed high-yielding and protein-containing Chinese cultivars (Kenworthy and Brim, 1979; Miller and Fehr, 1979; Burton and Brim, 1981; Junyi and Fehr, 1985; Burton et al., 1990; Roumet and Magnier, 1993; Qijian et al., 1996; Zhao et al., 2005, 2007; Deng et al., 2015; as reviewed by Nadeem et al., 2021).

## Conclusion

*Glycine max* (L.) Merr. bears cleistogamous flowers owing to its features of being cheap, affordable, and a rich source of proteins. Therefore, there are many challenges that are being faced to develop novel varieties, particularly, in soybean to meet the growing needs of food for the ever-growing population. Although soybean heterosis is not a new field, it is a potential area for future research. In spite of its importance, soybean is not gaining momentum in terms of research and scope. The addition of nonadditive genes is generally defined as heterosis. Heterosis helps in increasing the productivity of crops by around 15–50% and possesses the capacity to develop hybrids that contain better qualities than their parents. The most important factors that are commercially used in soybean production include yield, protein, and oil contents. So far, there is no commercial use of heterosis in soybean for the production of hybrids. However, in 2002, China released a hybrid variety of soybean. Since 1930, 14 reports showed that using 456 crosses, positive heterosis was found with the mid-parents ranging from 14 to 46% and high parents ranging from 4 to 34%. Therefore, there is a scope for the development of hybrids in soybean using appropriate methods of heterosis for obtaining superior hybrids. The male-sterile lines are important in understanding and studying the reproduction, cytogenetics, and genetic aspects of a crop to quantify its applicability in the production of hybrids at a commercial scale. The review aims to consider the soybean heterosis and use of MS so that it can be used in crop improvement programs to maximize the gain and, at the same time, ignite young researchers and students working on soybean to explore further as studies on proteins, amino acids, and oils of soybean are very scant.

## Author contributions

AyR and AmR conceptualized, resources, and data curated. AyR wrote the original draft and prepared the manuscript. AyR, AN, PB, RK, SM, BP, RV, SS, VK, SK, SKL, DR, and AmR contributed in revising and editing the manuscript. All the authors equally contributed to the manuscript revision and APC, read, and approved the submitted version.
